# Aberrant methylation of the M-type phospholipase A_2_ receptor gene in leukemic cells

**DOI:** 10.1186/1471-2407-12-576

**Published:** 2012-12-05

**Authors:** Mario Menschikowski, Uwe Platzbecker, Albert Hagelgans, Margot Vogel, Christian Thiede, Claudia Schönefeldt, Renate Lehnert, Graeme Eisenhofer, Gabriele Siegert

**Affiliations:** 1 Institut für Klinische Chemie und Laboratoriumsmedizin, Technische Universität Dresden, Fetscherstrasse 74, D-01307, Dresden, Germany; 2 Medizinische Klinik und Poliklinik I, Technische Universität Dresden, Dresden, Germany

**Keywords:** Phospholipase A_2_ receptor, DNA methylation, High resolution melting analysis, Leukemia

## Abstract

**Background:**

The M-type phospholipase A2 receptor (PLA2R1) plays a crucial role in several signaling pathways and may act as tumor-suppressor. This study examined the expression and methylation of the *PLA2R1* gene in Jurkat and U937 leukemic cell lines and its methylation in patients with myelodysplastic syndrome (MDS) or acute leukemia.

**Methods:**

Sites of methylation of the *PLA2R1* locus were identified by sequencing bisulfite-modified DNA fragments. Methylation specific-high resolution melting (MS-HRM) analysis was then carried out to quantify *PLA2R1* methylation at 5`-CpG sites identified with differences in methylation between healthy control subjects and leukemic patients using sequencing of bisulfite-modified genomic DNA.

**Results:**

Expression of PLA2R1 was found to be completely down-regulated in Jurkat and U937 cells, accompanied by complete methylation of *PLA2R1* promoter and down-stream regions; PLA2R1 was re-expressed after exposure of cells to 5-aza-2´-deoxycytidine. MS-HRM analysis of the *PLA2R1* locus in patients with different types of leukemia indicated an average methylation of 28.9% ± 17.8%, compared to less than 9% in control subjects. In MDS patients the extent of *PLA2R1* methylation significantly increased with disease risk. Furthermore, measurements of *PLA2R1* methylation appeared useful for predicting responsiveness to the methyltransferase inhibitor, azacitidine, as a pre-emptive treatment to avoid hematological relapse in patients with high-risk MDS or acute myeloid leukemia.

**Conclusions:**

The study shows for the first time that *PLA2R1* gene sequences are a target of hypermethylation in leukemia, which may have pathophysiological relevance for disease evolution in MDS and leukemogenesis.

## Background

Phospholipases A_2_ (sPLA_2_, phosphatide sn-2-acylhydrolases, EC 3.1.1.4) belong to the superfamily of phospholipases that catalyze the hydrolysis of the sn-2 ester bond in phospholipids generating free fatty acids and lysophospholipids [1]. Both reaction products may function as bioactive lipid mediators. In addition to functions dependent on their enzymatic activities, the group of secreted PLA_2_s can also act independently as receptor ligands. Most recently, sPLA_2_-IB and sPLA_2_-IIA have been identified as activators of different signaling pathways that are able to trigger cellular responses, such as cell proliferation, growth, differentiation and apoptosis [2-8].

To date, three types of potential binding sites for sPLA_2_ have been identified; the N (neuronal)-type receptor, the M (muscle)-type receptor (PLA2R1), and integrins αvβ3 and α4β1. The N-type receptor is mainly expressed in neuronal tissues and likely plays an important role in the mediation of neurotoxic effects of sPLA_2_s [9]. However, the binding affinities of sPLA_2_-IB and sPLA_2_-IIA to the N-type PLA_2_ receptor are low [10]. The M-type sPLA_2_ receptor was first identified in skeletal muscle, but is also expressed in other tissues [11]. The receptor has a high homology to the mannose-type receptor of macrophages, which is involved in the endocytosis of glycoproteins [12]. The M-type receptor is a type-1 180 kDa transmembrane glycoprotein containing extracellularly a cystein-reach domain, a fibronectin type II domain and eight carbohydrate recognition domains [10,13]. Recently, it was shown that patients with idiopathic membraneous nephropathy have antibodies against a conformation-dependent epitope in PLA2R1 [14]. Moreover, a high binding affinity of sPLA_2_-IIA to integrins of type αvβ3 and α4β1 was described in monocytic cells [6].

In the current study we analyzed the expression of the PLA2R1 in Jurkat and U937 leukemic cells and found for the first time a re-expression of the receptor after exposure of this cell lines to 5-aza-2´-deoxycytidine (5-aza-dC) suggesting that epigenetic mechanisms are involved in the regulation of this receptor. Based on this finding we further examined the methylation of the *PLA2R1* gene in tumor cell lines and in bone marrow aspirates of myelodysplastic syndrome (MDS) patients and compared the methylation of the *PLA2R1* gene in the peripheral blood of leukemic patients with those of healthy control subjects.

## Materials and methods

### Blood collections

Peripheral EDTA-blood samples were derived from 32 leukemic patients (20 females and 12 males) and 52 healthy individuals (34 females and 18 males). Median ages were 47.1 ± 28.6 years (range 2 to 78 years) in the leukemic patient group and 32.6 ± 16.3 years (range 1 to 82 years) in the healthy group. In addition, bone marrow aspirates were derived from 38 MDS patients (18 females and 20 males) with different IPSS classifications (12 patients with low-risk, 13 with intermediate-1-risk, five with intermediate-2-risk, and eight with high-risk classifications). Median ages were 68.8 ± 9.0 years (range 47 to 78 years) in the MDS patient group.

Furthermore, two EDTA-blood samples of 18 patients with high-risk MDS or AML, which were included in the clinical RELAZA trial [15] and who were treated with azacitidine (Vidaza; Celgene Corporation, Summit, NJ, USA; 75 mg/m^2^/day subcutaneously on days 1–7 of one course) were received for measurements of *PLA2R1* gene methylation at the beginning and end of the treatment. Finally, EDTA-blood samples of one patient (58 years old female) with AML, FAB M2, were received for serial measurements of *PLA2R1* gene methylation. After allogeneic hema-topoietic stem cell transplantation (HSCT) and with minimal residual disease (MRD), the patient underwent 3 courses of azacitidine treatment (75 mg/m^2^/day subcutaneously on days 1–7 per one course).

All patients provided written informed consent for the following studies and the use of the patient blood samples was approved by the Ethical Board of the University Hospital of Dresden. Patient demographics and disease characteristics are summarized in Additional file 1: Table S1, Additional file 2: Table S2 and Additional file 3: Table S3.

### Culture cell lines and incubation

Jurkat (human T lymphocyte acute leukemia), U937 (human hystiocytic lymphoma), Bv173 (human B-cell precursor leukemia), K-562 (human chronic myeloic leukemia in blast crisis), OC1-AML3 (human acute myeloid leukemia), KG-1A (human acute myeloid leukemia) and RPMI 8226 (human myeloma) cell lines were purchased from the German Collection of Microorganisms and Cell Cultures (DSMZ, Braunschweig, Germany). Cells were cultured in standard cell medium RPMI-1640 supplemented with 10% heat-inactivated fetal calf serum (FCS), 2 mM L-glutamine, 100 U/ml penicillin, and 100 μg/ml streptomycin at 37°C in a humidified atmosphere of 5% CO_2_. Exponentially growing cells were used in all experiments. In addition, genomic DNA from Raji (human B-cell leukemia), MCF7 (human mammary adenocarcinoma), and A431 (human melanoma) cell lines was purchased from BioCat GmbH (Heidelberg, Germany).

Jurkat and U937 leukemic cells were treated with 5-aza-2´-deoxycytidine (5-aza-dC), added to a final concentration of 1 μM. Cells were prepared at the end of the 3 day treatment period for analysis of PLA2R1- and TATA box binding protein (TBP)-specific mRNA.

### Extraction of genomic DNA and RNA

Genomic DNA was isolated from Jurkat, U937, Bv173, K-562, OC1-AML3, KG-1A and RPMI 8226 cell lines, peripheral blood samples, and bone marrow aspirates using a Blood & Cell Culture DNA Mini Kit from Qiagen GmbH (Hilden, Germany) and following the manufacturer`s instructions. RNA was isolated after lysis of Jurkat and U937 cells in TRI Reagent (Sigma-Aldrich, Deisenhofen, Germany) according to the manufacturer`s instructions.

### Quantitative RT-PCR analyses

Isolated RNA was converted to cDNA using the GeneAmp RNA-PCR Kit (PerkinElmer LAS GmbH, Jügesheim, Germany). For quantitative RT-PCR, portions of the reverse transcribed reaction products were then amplified for identification of PLA2R1 expression in comparison to those of TBP as a reference gene. Real-time RT-PCR was performed using Rotor-Gene Q (Qiagen GmbH) and Rotor Gene SYBR Green PCR kit according to manufacturer`s instructions. The applied primer pairs were 5`-CAG AAG AAA GGC AGT TCT GGA TTG-3` and 5`-AAA GCC ACA TCT CTG GCT CTG ATT-3´ for PLA2R1, giving PCR products with a length of 496 bp, and 5`-GAA TAT AAT CCC AAG CGG TTT G-3` and 5`-ACT TCA CAT CAC AGC TCC CC-3` for TBP amplifying products with 226 bp length. The primers were applied in a final concentration of 0.8 μM. The conditions for amplification were as follows: 40 courses at 95°C for 5 sec and 58°C for 10 sec. Amplified products were than analyzed by electrophoresis on agarose gels.

### Analysis of the promoter and down-stream regions of the PLA2R1 gene

MethPrimer software (http://www.urogene.org/methprimer) was used to analyze the proximal promoter and down-stream regions -700 to +1340 bp relative to exon 1 of *PLA2R1*[16], and thereby assess the presence of 5`-CpG islands in the promoter region.

### Bisulfite genomic sequencing

DNA methylation was analyzed using bisulfite-modified genomic DNA and by subsequent bidirectional sequencing. Aliquots of 500 ng of isolated genomic DNA were bisulfite modified [17-19] using the EpiTect Bisulfite Kit (Qiagen GmbH) according to manufacturer`s instructions. Three fragments expanding from −662 bp to +1275 bp relative to exon 1 were amplified by nested or semi-nested PCR. The applied primer pairs were 5`-TTT GTT GGT TAT TTG AAG GAG GA-3` and 5`-ACC ATC TAC CCA TCC CAA AA-3` as extrinsic and 5`-TAT ATT TTA GTT AGG GTT GTT TTA T-3` and 5`-TTC CTA CCT TTA AAA TAA AAA CAA A-3` as intrinsic primers for fragment 1 of *PLA2R1* (−662 bp to −107 bp from exon 1), giving PCR products with a length of 556 bp; for fragment 2 (−105 bp to +783 bp from exon 1) 5`-TTT GTT TTT ATT TTA AAG GTA GGA A-3` and 5`-ACC CTA TCT CAA AAA ACA AAC AA-3` as extrinsic and 5`-TTT TTG GGA TGG GTA GAT GG-3`and 5`-ACC TAA CTT AAA AAT CAC TCC TA-3` as intrinsic primers, giving PCR products with a length of 888 bp; and for fragment 3 (+761 bp to +1275 bp from exon 1) 5`-TAG GAG TGA TTT TTA AGT TAG GT-3` and 5`-CTC TCC TCC CTC TCT TTA CA-3` as extrinsic and 5`-TAG GAG TGA TTT TTA AGT TAG GT-3` and 5`-CAA CCT TCT AAA TCT CAT ATA TAA-3` as intrinsic primers in a semi-nested PCR, amplifying products with 515 bp length.

The primers were applied to a final concentration of 0.8 μM. The conditions for amplification were as follows: 15 courses with extrinsic primers at 94°C for 30 sec, 60-50°C as touch-down for 30 sec and 72°C for 1 min followed by 30 courses with intrinsic primers at 94°C for 30 sec, 62-52°C for 30 sec and 72°C for 1 min. Buffers and reagents were from GeneAmp Kit (PerkinElmer LAS GmbH). After amplification, PCR products were subjected to agarose gel electrophoresis to establish the purity of amplificates. Five ng of PCR products were then prepared for sequencing using ABI PRISM BigDye Terminator v3.1. Samples were purified using Agencourt CleanSEQ system (Beckman/Coulter Company; MS, USA) and sequenced using 3730 XL ABI/Hitachi. Sequences of bisulfite-treated genomic DNA were compared with those of untreated genomic DNA to verify the efficiency of bisulfite treatment. Ratios of cytosine:thymine and guanine:adenine residues in the forward or reverse sequencing were respectively calculated to assess the extent of methylation at different 5`-CpG sites.

### Methylation specific-high resolution melting (MS-HRM) analyses

MS-HRM analyses were carried out to quantify the extent of methylation in the distinct region −437 bp to −270 bp from exon 1 of the *PLA2R1* gene. These analyses were carried using Rotor-Gene Q (Qiagen GmbH) and the EpiTect MS-HRM PCR Kit according to manufacturer`s instructions. Bisulfite modified unmethylated and methylated standard DNA (Qiagen GmbH) were mixed giving samples with 0%, 10%, 20%, 30%, 50%, and 100% methylation ratios for calibration. A standard curve with known methylation ratios was included in each run. PCR was performed in 12.5 volumes. The applied primer pairs were 5`-GGG GTA AGG AAG GTG GAG AT-3` and 5`-ACA AAC CAC CTA AAT TCT AAT AAA CAC-3` giving PCR products with a length of 168 bp. The primers were applied at a final concentration of 0.8 μM. The conditions of amplification were as follows: 40 courses at 95°C for 10 seconds, 58°C for 30 seconds and 72°C for 15 sec. Immediately after PCR, products were analyzed by high resolution melting analysis with fluorescence measured during the linear temperature transition from 50-95°C at 0.01°C/second.

### Data analysis

Data were analyzed by two-tailed and unpaired Student`s *t-*test. Differences were considered significant at *p* < 0.05. Pearson Product Moment Correlation was applied to analyze the HRM values in relation to IPSS classification of MDS patients.

## Results

### Re-expression of PLA2R1 in human Jurkat and U937 leukemia cells

**Figure 1:**
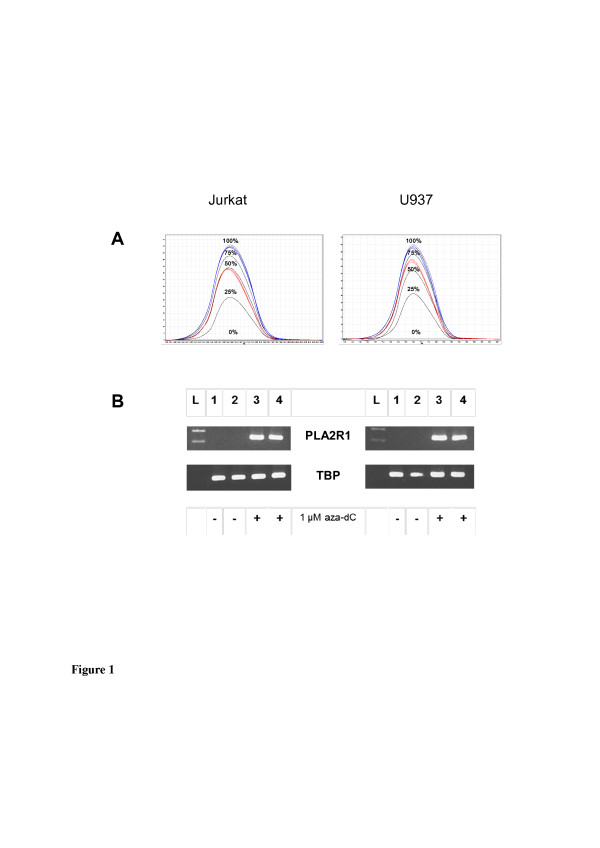
**Effects of 5-aza-2´-deoxycytidine (5-aza-dC) treatment on *PLA2R1 *methylation (A) and PLA2R1 expression (B) in Jurkat and U937 leukemic cells.** After exposure of cells to vehicle or 1 μM 5-aza-dC for 72 h mRNA and DNA were isolated. (**A**) MS-HRM analyses of *PLA2R1* methylations with a standard curve of 0%, 25%, 50%, 75%, and 100% methylation (in black lines) without (in blue) and with exposure (in red) of cells to the DNA demethylating agent, 5-aza-dC. Difference plots normalized to the 0%-methylated standard DNA sample are shown. (**B**) Agarose gel electrophoresis of RT-PCR amplificates of PLA2R1 in comparison to TBP-specific mRNA in Jurkat and U937 cells, with and without exposure to 5-aza-dC. Lanes 1 and 2; control cells (without addition of 5-aza-dC), lanes 3 and 4 represent cells treated respectively with 5-aza-dC, L; ladder. Analyses were performed in duplicates and results are representative of three independent experiments.

**Figure 2:**
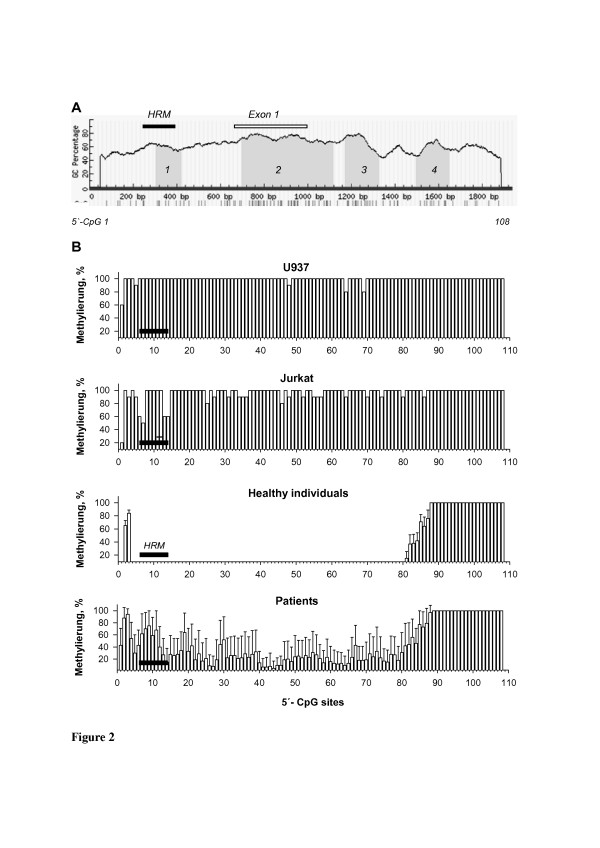
**5`-CpG islands and methylation of proximal promoter and down-stream regions of the *PLA2R1 *gene determined by sequencing of bisulfite modified genomic DNA.** (**A**) *PLA2R1* promoter and down-stream regions covering −700 to +1340 bp relative to exon 1 containing four 5`-CpG islands labelled in grey are shown; island-1, 115 bp length (−357 to −243 bp); island-2, 422 bp (+38 to +459); island-3, 157 bp (+512 to +668 bp), and island-4, 155 bp (+837 to +991 bp). Criteria used for 5`-CpG island prediction: island size >100 bp, GC percent >50.0, Minimum observed/expected >0.6. Horizontal black bar depicts the region expanding from −437 to −270 bp relative to exon 1 whose methylation was quantified using MS-HRM analysis. (**B**) Extents of methylation of distinct 5`-CpG sites in the *PLA2R1* promoter and down-stream region of U937 and Jurkat cells as well as *PLA2R1* methylations in DNA from blood of 10 healthy individuals and 16 leukemic patients (m*eans ± SDs)* determined by sequencing of bisulfite modified genomic DNA. Horizontal black bars show 5`-CpG sites analyzed by MS-HRM analyses.

Under control conditions, in Jurkat and U937 cells no PLA2R1-mRNA were detectable after RT-PCR and agarose gel electrophoresis (Figure 1A). Furthermore, no amplification occurred using real-time RT-PCR (the amplification efficiency was below 0.50). Simultaneously, the methylation of the *PLA2R1* gene averaged 100% in both cell lines analyzed by MS-HRM analyses (Figure 1B) and sequencing (Figure 2B) of bisulfite-modified genomic DNA. After exposure of cells to 5-aza-dC, however, significant PLA2R1 transcript levels were detectable both in the agarose gel electrophoresis (Figure 1A) and real-time RT-PCR (in Jurkat cells the ratio was 1.61x10(−E3) ± 0.36x10(−E3) with an efficiency of 1.56 and in U937 cells the ratio between PLA2R1/TBP mRNA levels averaged 1.21x10(−E4) ± 0.48x10(−E4) with an amplification efficiency of 1.74). In parallel, the *PLA2R1* gene methylation decreased from 100% to 50% in Jurkat cells and to 66% in U937 cells using MS-HRM analyses (Figure 1B).

### PLA2R1 gene 5`-CpG islands

Four distinct 5`-CpG islands were identified by Methprimer software-based analysis of the proximal promoter and down-stream regions covering -700 to +1340 bp from exon 1 of the *PLA2-R1* gene. The first island was located at −357 to −243 bp, the second at +38 to +459 bp, the third at +512 to +668 bp, and the fourth at +837 to +991 bp from exon 1 (Figure 2A).

### Methylation of the PLA2R1 gene in healthy individuals, leukemic cell lines and leukemic patients

Among healthy controls (N = 10), the methylation of 5`-CpG sites 2 (according to our nomenclature; -533 bp from exon 1), 3 (−522 bp), and 81 (+602 bp) to 108 (+1216 bp) was between 20% and 100%, whereas the 5`-CpG site 1 (−584 bp) and sites 4 to 80 (−473 bp to +586 bp from exon 1, respectively) were unmethylated (Figure 2B).

In contrast, in 15 patients with different types of leukemia (P1 to P3 and P5 to P16, Additional file 1: Table S1) a hypermethylation of the 5`-CpG sites 1 and 4 to 80 was found (Figure 2B). In one patient with AML, FAB M4 (P4), however, there was no detectable hypermethylation of this region (Additional file 1: Table S1).

In U937 and Jurkat cell lines a strong methylation of the 5`-CpG sites 1 and 4 to 80 in the *PLA2R1* gene was also identified (Figure 2B).

### PLA2R1 methylation quantified using MS-HRM analysis

Next, we used MS-HRM analyses to quantify *PLA2R1* methylation at 5`-CpG sites identified with most significant differences in methylation between healthy control subjects and leukemic patients. On the basis of sequencing data we selected a region between -437 to −270 bp relative to exon 1 of the *PLA2R1* gene (black line in Figure 2B) which covers the 5`-CpG sites 6–14 (according to our nomenclature; -415 bp to −298 bp from exon 1) and localizes in the proximal promoter of *PLA2R1*. This region showed the most significant differences in methylation between 10 healthy control subjects and 16 leukemic patients.

**Figure 3:**
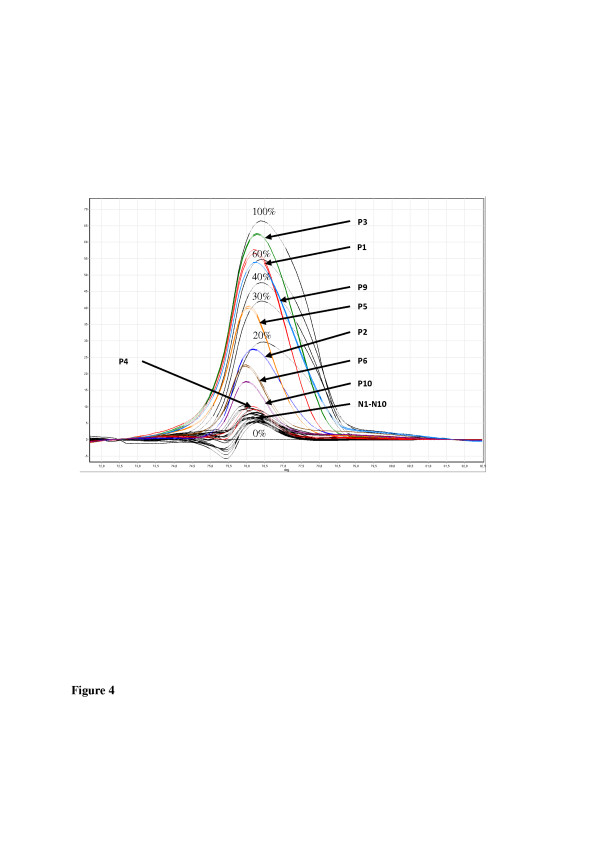
**Methylation of the *PLA2R1 *gene in DNA samples from blood of patients with leukemia in comparison to normal individuals.** MS-HRM analyses using RotorGene Q of amplified *PLA2R1* sequences covering 5`-CpG sites 6–14 (−437 bp to −270 bp from exon 1) after isolation and subsequent bisulfite modification of genomic DNA from normal subjects (N1-N10, black), and patients with leukemia (P1 to P6, P9, and P10; in colors, individual data are shown in Additional file 1: Table S1). Difference plots normalized to the 0%-methylated standard DNA sample and a standard curve with 0%, 20%, 30%, 40%, 60%, and 100% methylation ratios in black dotted lines are shown.

**Figure 4:**
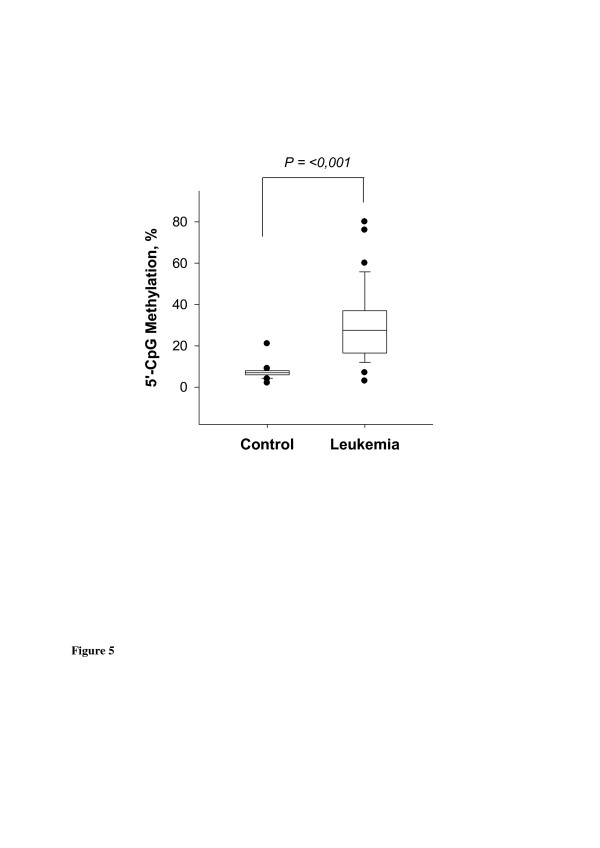
**Box-plots of *PLA2R1 *methylations in DNA from blood of healthy individuals (Controls, N = 52) and leukemic patients (Leukemia, N = 32).** MS-HRM analyses of amplified *PLA2R1* sequences (covering 5`-CpG sites 6–14, -437 bp to −270 bp from exon 1) after isolation and subsequent bisulfite modification of genomic DNA from blood samples were performed using RotorGene Q (Qiagen). Boxes within the plots represent the 25-75th percentiles. Median values are depicted as solid lines. Circles indicate outlier values outside of the 10th and 90th percentiles.

In Figure 3, examples of MS-HRM data from leukemic patients compared to normal subjects and in Table 1, MS-HRM data from analyzed cell lines are shown. The analysis of DNA from blood samples from 32 patients with leukemia and 52 normal individuals indicated respective rates of methylation of 28.9 ± 17.8% and 6.9 ± 2.5% (Figure 4, Table 2, and Additional file 1: Table S1). Patient P4, in who sequencing analysis indicated undetectable *PLA2R1* methylation in the region −473 bp and +586 bp, was found to have a 7% *PLA2R1* methylation by MS-HRM. In addition to patient P4, a second leukemic patient (P17, AML, FAB M0) showed no elevation in *PLA2R1* methylation (3% methylation degree, Additional file 1: Table S1). Among healthy subjects, there was one individual who showed a 21% level of methylation for the *PLA2R1* gene (N19, Additional file 1: Table S1).

**Table 1:** ***PLA2R1 *gene methylations in different human cell lines**

**Cell lines**	**classification**	***PLA2R1 *methylation/%**
Jurkat	human T cell leukemia	100
U937	human monocytic leukemia	100
Bv173	human B-cell precursor leukemia	100
Raji	human B cell leukemia	100
OC1-AML3	human akute myeloid leukemia	90
KG-1A	human acute myeloid leukemia	80
K-562	human CML in blast crisis	50
MCF7	human mammary adenocarcinoma	50
A431	human melanoma	48
RPMI-8226	human multiple myeloma	30

The degree of methylation was measured using MS-HRM analysis of bisulfite-modified genomic DNA.

**Table 2:** **Averaged *PLA2R1 *methylations in DNA from blood of normal individuals (Control, N = 52) and leukemic patients (Leukemia, N = 32) analysed by MS-HRM**

**Group**	**N**	**Methylation (mean ± SD)**
Control	52	6.9% ± 2.5%
Leukemia	32	28.9% ± 17.8% **

** p = <0.001 in comparison to controls

In the control group, one individual exhibited a *PLA2R1* methylation of 21% and in the leukemic group, two patients had methylations of 7% and 3% (patient details see in Additional file 1: TableS1). The 95^th^ percentile value usable as cut-off averaged 9%.

**Figure 5:**
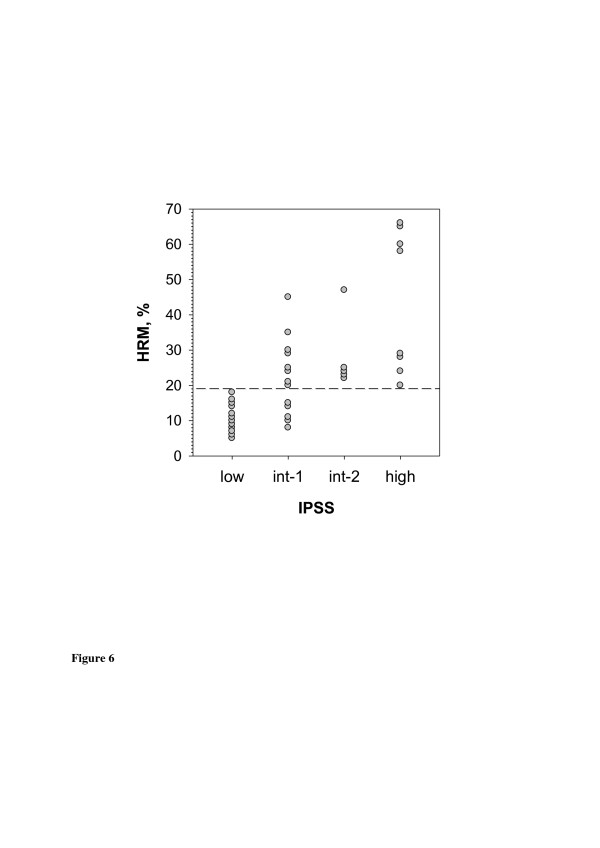
**Methylation of the *PLA2R1 *gene in bone marrow aspirates of MDS patients in dependence on IPSS classification.** After isolation and subsequent bisulfite modification of genomic DNA from bone marrow aspirates of MDS patients (N = 38) methylations of amplified *PLA2R1* sequences covering 5`-CpG sites 6–14 (−437 bp to −270 bp from exon 1) were analyzed using MS-HRM (HRM,%). Low, low risk; int-1, intermediate-1-risk; int-2, intermediate-2-risk; and high, high-risk classifications.

Among the group of MDS patients, the melt curves of *PLA2R1* amplificates from bone marrow aspirates differed considerably with increased IPSS classification. In particular, the level of *PLA2R1* methylation increased with the disease stage (Additional file 4: Figure S1). Consistent with this, MS-HRM values of *PLA2R1* methylation correlated with the IPSS classification (r = 0.661, p < 0.00001). While *PLA2R1* methylation was below 19% in all of low-risk patients, the MS-HRM values were only partially below this degree in intermediate-1-risk patients and significantly higher in the groups of intermediate-2 and high-risk patients (Figure 5 and Additional file 2: Table S2).

### PLA2R1 methylations in MDS and AML patients during azacitidine treatment

In the group of 18 high-risk MDS and AML patients treated with azacitidine as methyltransferase inhibitor, six patients had *PLA2R1* methylation degrees above 9% (Additional file 3: Table S3). In course of treatment with azacitidine, three of these patients (P73, P87 and P88) showed decreased methylation levels and can be classified as responders. In contrast, one patient (P86) showed increased methylation level in blood leukocytes after treatment in comparison to those at the beginning of treatment. This patient together with two patients whose methylation degrees were unchanged after azacitidine treatment, can be classified as non-responders (Additional file 3: Table S3). Among patients with *PLA2R1* methylation of 9% or below, four patients showed, respectively decreased (P75, P76, P79 and P80) or increased methylations (P74, P78, P81 and P84) after azacitidine treatment (Additional file 3: Table S3).

**Figure 6:**
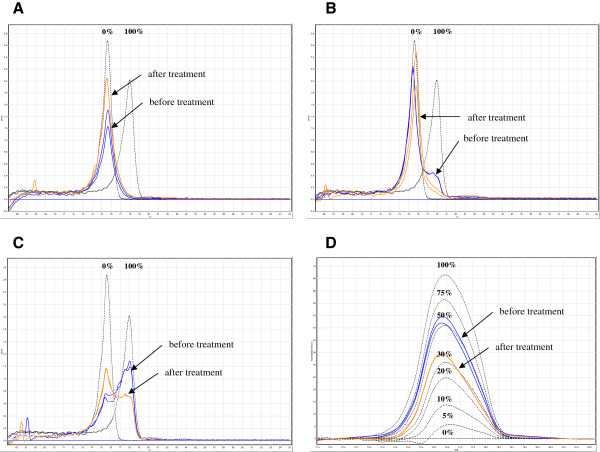
**Methylation of *PLA2R1 *in DNA from the blood of a patient with AML after hematopoietic stem cell transplantation and minimal residual disease during three courses with azacitidine treatment.** (**A**) Melt curve analyses of bisulfite-modified genomic DNA at the start (blue) and end (orange) of the first course of treatment, (**B**) at the start (blue) and end (orange) of the second course of treatment, and (**C**) at the start (blue) and end (orange) of the third course of treatment. (**D**) MS-HRM analyses at the start (blue) and end (orange) of the third course of azacitidine treatment. Melt curves of unmethylated (0%) and methylated (100%) standard DNA samples (**A**-**C**) and a standard curve with 0%, 5%, 10%, 20%, 30%, 50%, 75%, and 100% methylation ratios (**D**) are shown in black. (**A**-**C**); fluorescence dF/dT and (**D**) difference plot normalized minus 0. Analyses were performed in duplicates and results are representative of three independent measurements.

Furthermore, we analyzed the *PLA2R1* methylation in a patient with AML (FAB M2) following allogeneic HSCT, who had minimal residual disease (MRD) and underwent pre-emptive treatment with azacitidine to avoid haematological relapse (Additional file 5: Table S4). Methylation of the selected *PLA2R1* region was below 9% at the start and end of the first course of treatment; normal melt curves of amplicons were also found (Figure 6A). At the beginning of second treatment course, a moderate increase of *PLA2R1* methylation at 12% was measured, which decreased to 9% at the end of the treatment course (Additional file 5: Table S4). Melt curve analysis indicated a minor fraction of completely methylated *PLA2R1* at the beginning of this treatment course, which completely disappeared after treatment (Figure 6B). Twenty-two days later at the beginning of course 3, the methylation of the tested *PLA2R1* region in DNA from blood averaged 50% (Figure 6C and 6D) and 9% blasts were detectable (Additional file 5: Table S4). A similar distribution of unmethylated and methylated DNA was measured in a bone marrow DNA sample using MS-HRM and melt curve analyses at the same time point (data not shown). At the end of course 3 with azacitidine treatment, the methylation of *PLA2R1* in DNA from blood decreased, but remained at 35% (Figure 6D). At the same time the MRD, as detected by CD34-donor chimerism, had further increased suggesting no sufficient response to pre-emptive azacitidine treatment. Shortly later the patient developed overt haematological relapse.

## Discussion

This study establishes for the first time that epigenetic mechanisms are involved in the regulation of PLA2R1 expression in leukemic cells. On the basis of two previously published studies the observed epigenetic down-regulation of PLA2R1 in leukemic cells may have pathophysiological relevance. First, it was shown that secreted phospholipases A_2_ (types IIA and IB) induce cellular senescence through a p53-dependent pathway [20]. Second, the inhibition of the cell growth induced by secreted phospholipase A_2_ (type IIA) overexpression is reverted in PLA2R1-depleted cells [21]. Using a retroviral short hairpin RNA library targeting about 8,000 human genes the authors of the latter study identified the PLA2R1, along with four other genes, to play a crucial role in senescent primary human fibroblasts. Moreover, they demonstrated that the effect of PLA2R1 down-regulation is not limited to primary human fibroblasts, but is also present in other human cells such primary human mammary epithelial cells [21].

The above findings serve to emphasize the possible role of *PLA2R1* gene hypermethylation in human blood cells of leukemic patients. The process of senescence is seen as a tumor suppression mechanism preventing malignant transformation. Inversely, an escape from senescence can lead to a progression of malignancy [21-24]. Therefore, in future studies it will be interesting to define cell types, in which the *PLA2R1* gene is hypermethylated and whether in these cells the hypermethylation is connected with *PLA2R1* silencing.

Additionally, the study of MDS patients demonstrates that *PLA2R1* gene methylation correlates with IPSS classification and may be useful as an additional biomarker for risk stratification. Our data confirm previous work demonstrating increased methylation with MDS disease stage, suggesting that hypermethylation of certain tumor suppressor genes is a mechanism of disease evolution in leukemogenesis [25].

A further question arising from our study is whether the extent of *PLA2R1* methylation can be used as a biomarker for stratifying patients for treatment of leukemia. An early indicator of a potential response to treatment could be, for example, particularly useful when initiating treatment with methyltransferase inhibitors such as azacitidine. The data received in a first group of patient who were treated with azacitidine show that a significantly decrease in extent of *PLA2R1* methylation after one course of 5-aza-dC treatment can be measured in a subgroup of patients, whereas in other patients the methylation was unchanged or even increased. A similar effect of demethylating treatment on gene methylation was described in case of *phosphoinositide-phospholipase C-beta1*[26]. These suggest that the *PLA2R1* gene may function as a possible target to monitor the efficiency of demethylating therapy. In addition, we show in a patient with AML after HSCT, how melt curve analyses of amplified *PLA2R1* sequences may indicate a recurrence or progression of the disease. For this reason further studies seem warranted to follow the effects of demethylating therapies on *PLA2R1* methylation in a larger number of leukemic patients.

In addition to diagnosis and control of methylation-related therapies the observation that inhibition of DNA methylation can restore the PLA2R1 expression may have important implications for development of novel strategies to treat leukemia. There is growing evidence that anti-oncogenic and pro-apoptotic properties of sPLA_2_ isozymes may have importance for defective pathways of apoptosis in tumorigenesis [27-31]. However, little is known about the biological function and regulation of PLA2R1 expression as potential binding site of sPLA2s. In PLA2R1-deficient mice lower blood levels of TNF-α- and IL-1β were found after lipopolysaccharide application which was connected with a relative resistance to lipopolysaccharide-induced lethality. Therefore, a pro-inflammatory function was postulated for PLA2R1 [32]. Furthermore, an endocytosis of PLA2R1 was described similar to the LDL-receptor associated with lysosomal degradation of bound sPLA2s suggesting that the receptor plays a crucial role in the regulation of sPLA2 activities during inflammation [10,33]. On the basis of the finding that the PLA2R1 can be restored in Jurkat and U937 cells by demethylating treatment, new opportunities are opened to analyze the effects of the receptor re-expression on senescence and apoptosis. These studies may be a source for developing alternative strategies for leukemic treatment using the PLA2R1 as target.

## Conclusions

The data of this study indicate that in Jurkat and U937 leukemic cells the inhibition of methylation by 5-aza-dC re-expresses PLA2R1 and that in MDS and leukemic patients, in contrast to healthy individuals, *PLA2R1* promoter sequences are hypermethylated. This suggests that, in addition to chromosome aberrations and gene mutations, aberrant methylation plays a crucial role in MDS and its evolution to AML. The study also shows that the determination of *PLA2R1* methylation by melt curve and MS-HRM analyses may be useful for risk stratification of MDS and AML patients and therapeutic control of leukemia using methylation inhibitors.

## Abbreviations

AML: Acute myeloid leukemia; 5-aza-dC: 5-aza-2´-deoxycytidine; CMML-1: Chronic myelomonocytic leukemia; EDTA: Ethylenediaminetetraacetic acid; FAB: French-American-British; FCS: Fetal calf serum; HRM: High resolution melting; HSCT: Hematopoietic stem cell transplantation; IPSS: International Prognostic Scoring System; MDS: Myelodysplastic syndrome; MRD: Minimal residual disease; MS-HRM: Methylation specific-high resolution melting; PLA2R1: M-type phospholipase A_2_ receptor; RAEB: Refractory anemia with excess blasts; RAEB-t: Refractory anemia with excess blasts in transformation; RCMD: Refractory cytopenia with multilineage dysplasia; RCMD-RS: Refractory cytopenia with multilineage dysplasia and ringed sideroblasts; RCUD: Refractory cytopenia with unilineage dysplasia; RT-qPCR: Reverse transcription quantitative polymerase chain reaction; sAML: Secondary acute myeloid leukemia; sPLA_2_-IB: Secreted phospholipase A_2_ of group IB; sPLA_2_-IIA: Secreted phospholipase A_2_ of group IIA; TBP: TATA box binding protein.

## Competing interest

The authors declare that they have no competing interests.

## Authors’ contributions

MM and UP provided concept and design of the study; UP, CT and CS were involved in sample collection and data retrieval; MM, AH, MV RL performed the analyses; MM wrote the manuscript; UP, AH, CT, GE and GS provided drafting of the article; and all authors approved the final submitted manuscript.

## Pre-publication history

The pre-publication history for this paper can be accessed here:

http://www.biomedcentral.com/1471-2407/12/576/prepub

## Supplementary Material

Additional file 1 **Table S1.** Characteristics of patients analyzed for *PLA2R1* methylation using MS-HRM analysis of bisulfite-modified genomic DNA. Click here for file

Additional file 2 **Table S2.** Characteristics of MDS patients with different IPSS classifications. The degree of *PLA2R1* methylation shown was measured using MS-HRM analysis of bisulfite-modified genomic DNA from bone marrow aspirates. CMML-1, chronic myelomonocytic leukemia; RCMD, refractory cytopenia with multilineage dysplasia; RCMD-RS, refractory cytopenia with multilineage dysplasia and ringed sideroblasts; RAEB, refractory anemia with excess blasts; RCUD, refractory cytopenia with unilineage dysplasia; RAEB-t, refractory anemia with excess blasts in transformation; sAML, secondary acute myeloid leukemia. Click here for file

Additional file 3 **Table S3.** Characteristics of patients treated with azacitidine. The degree of *PLA2R1* methylation shown was measured using MS-HRM analysis of bisulfite-modified genomic DNA from blood samples. RCMD, refractory cytopenia with multilineage dysplasia; RAEB, refractory anemia with excess blasts; RAEB-t, refractory anemia with excess blasts in transformation. Click here for file

Additional file 4 **Figure S1.** Melt curve analyses of bisulfite-modified and amplified *PLA2R1* gene sequences in bone marrow aspirates of MDS patients. After isolation and subsequent bisulfite modification of genomic DNA from bone marrow aspirates melt properties of amplified *PLA2R1* sequences covering 5`-CpG sites 6–14 (−437 bp to −270 bp from exon 1) were analyzed using RotorGene Q. P34; a 74 years old male patient with RCMD and low-risk according to IPSS classification, P57; a 69 years old male patient with RAEB-2 and intermediate-1-risk, P66; a 69 years old female patient with RAEB-2 and intermediate-2-risk, and P69; a 56 years old male patient with AML and high-risk classification. Individual data of MDS patients are summarized in Additional file 2: Table S2. Melt curves of amplified genomic DNA from MDS patients (in colors) and those of unmethylated (0%) and methylated (100%) standard DNA samples (in black) are shown. Fluorescence dF/dT were measured. Analyses were performed in duplicates and results are representative of three independent measurements. Click here for file

Additional file 5 **Table S4.** MS-HRM analyses of bisulfite-modified genomic DNA isolated from peripheral blood samples of a 58 years old female patient with minimal residual disease. After treatment of AML, FAB M2, by allogeneic hematopoietic stem cell transplantation three years ago the patient underwent a pre-emptive treatment with azacitidine to avoid haematological relapse. Amounts of leukocytes, blasts, and *PLA2R1* methylation degrees during treatment measured using MS-HRM analysis are summerized. n; normal melt curve without methylated DNA fraction, p; pathological melt curve with methylated DNA fraction in addition to unmethylated DNA fraction. Click here for file
