# Supplementary material for: Aberrant methylation of the M-type phospholipase A2 receptor gene in leukemic cells
Source: BMC Cancer. 2012 Dec 5;12:576. doi: 10.1186/1471-2407-12-576 (PMC3561142; doi:10.1186/1471-2407-12-576)
Supplement: Additional file 1 — Table S1. Characteristics of patients analyzed for PLA2R1 methylation using MS-HRM analysis of bisulfite-modified genomic DNA. [file 1471-2407-12-576-S1.docx]

**P34**

**P57**

**P66**

**P69**

0%

100%

0%

100%

0%

100%

0%

100%

**Supplementary Figure 1**
