# Supplementary material for: Aberrant methylation of the M-type phospholipase A2 receptor gene in leukemic cells
Source: BMC Cancer. 2012 Dec 5;12:576. doi: 10.1186/1471-2407-12-576 (PMC3561142; doi:10.1186/1471-2407-12-576)
Supplement: Additional file 2 — Table S2. Characteristics of MDS patients with different IPSS classifications. The degree of PLA2R1 methylation shown was measured using MS-HRM analysis of bisulfite-modified genomic DNA from bone marrow aspirates. CMML-1, chronic myelomonocytic leukemia; RCMD, refractory cytopenia with multilineage dysplasia; RCMD-RS, refractory cytopenia with multilineage dysplasia and ringed sideroblasts; RAEB, refractory anemia with excess blasts; RCUD, refractory cytopenia with unilineage dysplasia; RAEB-t, refractory anemia with excess blasts in transformation; sAML, secondary acute myeloid leukemia. [file 1471-2407-12-576-S2.docx]

**Supplementary Table 1.** Characteristics of patients analyzed for *PLA2R1* methylation using MS-HRM analysis of bisulfite-modified genomic DNA.

Leukemic group:

| **No.** | **ID** | | **Age/ years** | **Gender** | **Disease** | ***PLA2R1* methylation, %** |
| --- | --- | --- | --- | --- | --- | --- |
| 1 | P1 | | 42 | f | AML, FAB M1 | 60 |
| 2 | P2 | | 77 | f | MDS, sAML,  FAB M1 | 19 |
| 3 | P3 | | 76 | m | AML, FAB M1 | 80 |
| 4 | P4 | | 66 | f | AML, FAB M4 | 7 |
| 5 | P5 | | 5 | f | c-ALL | 30 |
| 6 | P6 | | 74 | m | AML, FAB M1 | 16 |
| 7 | P7 | | 78 | m | AML, FAB M2 | 27 |
| 8 | P8 | | 68 | m | AML, FAB M1 | 12 |
| 9 | P9 | | 71 | f | AML, FAB M1 | 43 |
| 10 | P10 | | 2 | f | pre-B-ALL | 15 |
| 11 | P11 | | 4 | m | c-ALL | 16 |
| 12 | P12 | | 77 | f | Pro-lymphocytic leukemia | 13 |
| 13 | P13 | | 67 | f | AML, FAB M1 | 28 |
| 14 | P14 | | 49 | f | AML, FAB M5A | 37 |
| 15 | P15 | | 74 | f | AML, FAB M6 | 37 |
| 16 | P16 | | 56 | m | AML, FAB M1 | 18 |
| 17 | | P17 | 61 | f | AML, FAB M0 | 3 |
| 18 | | P18 | 6 | m | pre-B-ALL | 18 |
| 19 | | P19 | 8 | m | c-ALL | 21 |
| 20 | | P20 | 66 | f | B-ALL | 18 |
| 21 | | P21 | 47 | f | B-CLL | 28 |
| 22 | | P22 | 69 | m | B-CLL | 24 |
| 23 | | P23 | 29 | f | pre-T-ALL | 31 |
| 24 | | P24 | 20 | m | AML, FAB M3 | 38 |
| 25 | | P25 | 2 | f | c-ALL | 12 |
| 26 | | P26 | 61 | m | AML, FAB M5A | 20 |
| 27 | | P27 | 73 | f | AML, FAB M5A | 28 |
| 28 | | P28 | 51 | f | AML, FAB M1 | 35 |
| 29 | | P29 | 4 | m | c-ALL | 40 |
| 30 | | P30 | 2 | f | AML, FAB M7 | 76 |
| 31 | | P31 | 72 | f | AML, FAB M1 | 29 |
| 32 | | P32 | 51 | f | AML, FAB M4 | 46 |

Control group (healthy individuals):

| **No.** | **ID** | **Age/ years** | **Gender** | ***PLA2R1 m*ethylation, %** |
| --- | --- | --- | --- | --- |
| 1 | N1 | 38 | f | 7 |
| 2 | N2 | 26 | m | 8 |
| 3 | N3 | 15 | m | 6 |
| 4 | N4 | 22 | m | 8 |
| 5 | N5 | 47 | m | 6 |
| 6 | N6 | 53 | f | 8 |
| 7 | N7 | 1 | f | 6 |
| 8 | N8 | 24 | f | 6 |
| 9 | N9 | 24 | f | 8 |
| 10 | N10 | 57 | f | 6 |
| 11 | N11 | 20 | m | 6 |
| 12 | N12 | 43 | m | 4 |
| 13 | N13 | 48 | m | 6 |
| 14 | N14 | 76 | m | 8 |
| 15 | N15 | 45 | m | 8 |
| 16 | N16 | 72 | f | 8 |
| 17 | N17 | 82 | m | 4 |
| 18 | N18 | 34 | f | 6 |
| 19 | N19 | 46 | f | 21 |
| 20 | N20 | 25 | f | 6 |
| 21 | N21 | 23 | m | 8 |
| 22 | N22 | 21 | f | 8 |
| 23 | N23 | 22 | f | 8 |
| 24 | N24 | 20 | f | 7 |
| 25 | N25 | 24 | f | 9 |
| 26 | N26 | 32 | m | 9 |
| 27 | N27 | 29 | f | 6 |
| 28 | N28 | 24 | f | 6 |
| 29 | N29 | 21 | m | 6 |
| 30 | N30 | 19 | f | 7 |
| 31 | N31 | 19 | f | 2 |
| 32 | N32 | 18 | f | 6 |
| 33 | N33 | 25 | f | 7 |
| 34 | N34 | 26 | f | 6 |
| 35 | N35 | 26 | f | 6 |
| 36 | N36 | 33 | m | 6 |
| 37 | N37 | 25 | f | 5 |
| 38 | N38 | 29 | f | 9 |
| 39 | N39 | 27 | m | 7 |
| 40 | N40 | 29 | m | 8 |
| 41 | N41 | 23 | f | 8 |
| 42 | N42 | 23 | f | 6 |
| 43 | N43 | 19 | f | 6 |
| 44 | N44 | 28 | m | 8 |
| 45 | N45 | 17 | f | 4 |
| 46 | N46 | 20 | f | 8 |
| 47 | N47 | 57 | f | 7 |
| 48 | N48 | 43 | f | 7 |
| 49 | N49 | 39 | m | 8 |
| 50 | N50 | 55 | f | 7 |
| 51 | N51 | 49 | f | 5 |
| 52 | N52 | 33 | f | 4 |
