# Supplementary material for: Aberrant methylation of the M-type phospholipase A2 receptor gene in leukemic cells
Source: BMC Cancer. 2012 Dec 5;12:576. doi: 10.1186/1471-2407-12-576 (PMC3561142; doi:10.1186/1471-2407-12-576)
Supplement: Additional file 3 — Table S3. Characteristics of patients treated with azacitidine. The degree of PLA2R1 methylation shown was measured using MS-HRM analysis of bisulfite-modified genomic DNA from blood samples. RCMD, refractory cytopenia with multilineage dysplasia; RAEB, refractory anemia with excess blasts; RAEB-t, refractory anemia with excess blasts in transformation. [file 1471-2407-12-576-S3.docx]

**Supplementary Table 2.** **Characteristics of MDS patients with different IPSS classifications.** The degree of *PLA2R1* methylation shown was measured using MS-HRM analysis of bisulfite-modified genomic DNA from bone marrow aspirates.

| **No.** | **ID** | **Age** | **Gender** | **MDS WHO subtype (FAB)** | **IPSS** | ***PLA2R1 m*ethylation, %** |
| --- | --- | --- | --- | --- | --- | --- |
| 1 | P33 | 67 | M | CMML-1 | low-risk | 5 |
| 2 | P34 | 74 | M | RCMD | low-risk | 6 |
| 3 | P35 | 59 | M | RCMD | low-risk | 8 |
| 4 | P36 | 71 | M | CMML-1 | intermediate-1-risk | 8 |
| 5 | P37 | 53 | M | RCMD | low-risk | 8 |
| 6 | P38 | 67 | F | RCMD | low-risk | 8 |
| 7 | P39 | 53 | M | RCMD | low-risk | 10 |
| 8 | P40 | 73 | F | RCMD | intermediate-1-risk | 10 |
| 9 | P41 | 60 | F | MDS  del(5q) | intermediate-1-risk | 10 |
| 10 | P42 | 61 | F | RCMD | low-risk | 10 |
| 11 | P43 | 60 | F | RCMD-RS | low-risk | 11 |
| 12 | P44 | 76 | F | MDS  del(5q) | low-risk | 14 |
| 13 | P45 | 74 | M | RCMD | intermediate-1-risk | 14 |
| 14 | P46 | 72 | F | MDS  del(5q) | low-risk | 15 |
| 15 | P47 | 59 | F | RCMD | intermediate-1-risk | 15 |
| 16 | P48 | 53 | M | RCMD | low-risk | 16 |
| 17 | P49 | 48 | M | RCMD | low-risk | 18 |
| 18 | P50 | 47 | F | RAEB-1 | intermediate-1-risk | 20 |
| 19 | P51 | 78 | M | RAEB-2 | high-risk | 20 |
| 20 | P52 | 53 | M | RCUD | intermediate-1-risk | 21 |
| 21 | P53 | 62 | F | sAML (RAEB-t) | intermediate-2-risk | 22 |
| 22 | P54 | 67 | M | sAML (RAEB-t) | intermediate-2-risk | 22 |
| 23 | P55 | 61 | F | RCMD | intermediate-2-risk | 22 |
| 24 | P56 | 71 | F | RCMD | high-risk | 24 |
| 25 | P57 | 69 | M | RAEB-2 | intermediate-1-risk | 24 |
| 26 | P58 | 77 | M | RCMD | intermediate-1-risk | 25 |
| 27 | P59 | 72 | F | RAEB-2 | intermediate-2-risk | 25 |
| 28 | P60 | 69 | M | sAML (RAEB-t) | high-risk | 28 |
| 29 | P61 | 77 | M | sAML (RAEB-t) | high-risk | 28 |
| 30 | P62 | 62 | F | RCMD | intermediate-1-risk | 29 |
| 31 | P63 | 47 | F | RCMD | intermediate-1-risk | 30 |
| 32 | P64 | 63 | M | RCMD | intermediate-1-risk | 35 |
| 33 | P65 | 70 | M | RCMD | intermediate-1-risk | 45 |
| 34 | P66 | 69 | F | RAEB-2 | intermediate-2-risk | 47 |
| 35 | P67 | 65 | M | sAML (RAEB-t) | high-risk | 58 |
| 36 | P68 | 59 | F | sAML (RAEB-t) | high-risk | 60 |
| 37 | P69 | 56 | M | sAML  (FAB M1) | high-risk | 65 |
| 38 | P70 | 52 | F | sAML (RAEB-t) | high-risk | 65 |

CMML-1, chronic myelomonocytic leukemia; RCMD, refractory cytopenia with multilineage dysplasia; RCMD-RS, refractory cytopenia with multilineage dysplasia and ringed sideroblasts; RAEB, refractory anemia with excess blasts; RCUD, refractory cytopenia with unilineage dysplasia; RAEB-t, refractory anemia with excess blasts in transformation; sAML, secondary acute myeloid leukemia.
