# Supplementary material for: Aberrant methylation of the M-type phospholipase A2 receptor gene in leukemic cells
Source: BMC Cancer. 2012 Dec 5;12:576. doi: 10.1186/1471-2407-12-576 (PMC3561142; doi:10.1186/1471-2407-12-576)
Supplement: Additional file 4 — Figure S1. Melt curve analyses of bisulfite-modified and amplified PLA2R1 gene sequences in bone marrow aspirates of MDS patients. After isolation and subsequent bisulfite modification of genomic DNA from bone marrow aspirates melt properties of amplified PLA2R1 sequences covering 5`-CpG sites 6–14 (−437 bp to −270 bp from exon 1) were analyzed using RotorGene Q. P34; a 74 years old male patient with RCMD and low-risk according to IPSS classification, P57; a 69 years old male patient with RAEB-2 and intermediate-1-risk, P66; a 69 years old female patient with RAEB-2 and intermediate-2-risk, and P69; a 56 years old male patient with AML and high-risk classification. Individual data of MDS patients are summarized in Additional file 2: Table S2. Melt curves of amplified genomic DNA from MDS patients (in colors) and those of unmethylated (0%) and methylated (100%) standard DNA samples (in black) are shown. Fluorescence dF/dT were measured. Analyses were performed in duplicates and results are representative of three independent measurements. [file 1471-2407-12-576-S4.docx]

**Supplementary Table 3.** **Characteristics of patients treated with azacitidine.** The degree of *PLA2R1* methylation shown was measured using MS-HRM analysis of bisulfite-modified genomic DNA from blood samples.

| **No.** | **ID** | **Age** | **Gender** | **MDS WHO subtype** | **Days (start/end of one course)** | **Cycle** | ***PLA2R1 m*ethylation (start/end of the treatment)** |
| --- | --- | --- | --- | --- | --- | --- | --- |
| 1 | P71 | 62 | M | AML, FAB M2 | 1/ 7 | 1 | 6%/ 6% |
| 2 | P72 | 72 | F | RAEB-2 | 1/ 7 | 1 | 8%/ 8% |
| 3 | P73 | 87 | F | AML, FAB M4 | 1/ 7 | 1 | 36%/ 16% |
| 4 | P74 | 69 | F | AML, FAB M1 | 1/ 7 | 1 | 4%/ 7% |
| 5 | P75 | 68 | M | AML, FAB M0 | 1/ 7 | 3 | 5%/ 3% |
| 6 | P76 | 70 | M | AML, FAB M1 | 1/ 7 | 1 | 3%/ 2% |
| 7 | P77 | 58 | F | AML, FAB M2 | 1/ 7 | 1 | 3%/ 3% |
| 8 | P78 | 79 | F | RAEB-t | 1/ 7 | 5 | 4%/ 6% |
| 9 | P79 | 61 | M | AML, FAB M1 | 1/ 7 | 15 | 9%/ 7% |
| 10 | P80 | 66 | F | RAEB-2 | 1/ 7 | 1 | 9%/ 8% |
| 11 | P81 | 61 | M | AML, FAB M2 | 1/ 5 | 14 | 9%/ 11% |
| 12 | P82 | 70 | F | RAEB-1 | 1/ 5 | 6 | 11%/ 11% |
| 13 | P83 | 60 | F | RAEB-1 | 1/ 5 | 4 | 10%/ 10% |
| 14 | P84 | 65 | M | AML, FAB M6 | 1/ 5 | 8 | 9%/ 11% |
| 15 | P85 | 66 | M | RCMD | 1/ 7 | 2 | 6%/ 6% |
| 16 | P86 | 53 | M | acute biphenotypic leukemia | 1/ 5 | 1 | 12%/ 19% |
| 17 | P87 | 77 | M | RAEB-2 | 1/ 7 | 1 | 13%/ 7% |
| 18 | P88 | 66 | F | AML, FAB M2 | 1/ 7 | 1 | 30%/ 23% |

RCMD, refractory cytopenia with multilineage dysplasia; RAEB, refractory anemia with excess blasts; RAEB-t, refractory anemia with excess blasts in transformation.
