# Supplementary material for: Aberrant methylation of the M-type phospholipase A2 receptor gene in leukemic cells
Source: BMC Cancer. 2012 Dec 5;12:576. doi: 10.1186/1471-2407-12-576 (PMC3561142; doi:10.1186/1471-2407-12-576)
Supplement: Additional file 5 — Table S4. MS-HRM analyses of bisulfite-modified genomic DNA isolated from peripheral blood samples of a 58 years old female patient with minimal residual disease. After treatment of AML, FAB M2, by allogeneic hematopoietic stem cell transplantation three years ago the patient underwent a pre-emptive treatment with azacitidine to avoid haematological relapse. Amounts of leukocytes, blasts, and PLA2R1 methylation degrees during treatment measured using MS-HRM analysis are summerized. n; normal melt curve without methylated DNA fraction, p; pathological melt curve with methylated DNA fraction in addition to unmethylated DNA fraction. [file 1471-2407-12-576-S5.docx]

**Supplementary Table 4.** **MS-HRM analyses of bisulfite-modified genomic DNA isolated from peripheral blood samples of a 58 years old female patient with minimal residual disease.** After treatment of AML, FAB M2, by allogeneic hematopoietic stem cell transplantation three years ago the patient underwent a pre-emptive treatment with azacitidine to avoid haematological relapse. Amounts of leukocytes, blasts, and *PLA2R1* methylation degrees during treatment measured using MS-HRM analysis are summerized. n; normal melt curve without methylated DNA fraction, p; pathological melt curve with methylated DNA fraction in addition to unmethylated DNA fraction.

| **Day no.** | **Course no.** | **leukocytes,**  **Gpt/l** | **blasts,**  **%** | ***PLA2R1***  ***m*ethylation** | ***PLA2R1***  **melt curve** |
| --- | --- | --- | --- | --- | --- |
| 0 | Course 1, day 1 | 5.7 | 0 | 8% | n |
| +7 | Course 1, day 7 | 6.4 | 0 | 8% | n |
| +37 | Course 2, day 1 | 11.7 | 1 | 12% | p |
| +44 | Course 2, day 7 | 7.3 | 0 | 9% | n |
| +66 | Course 3, day 1 | 2.6 | 9 | 50% | p |
| +73 | Course 3, day 7 | 1.9 | 0 | 35% | p |
